# Exploring challenges and enablers for community pharmacists using electronic prescriptions (Wasfaty) in Makkah region, Saudi Arabia: a qualitative study using the theoretical domains framework

**DOI:** 10.3389/fmed.2024.1487852

**Published:** 2024-11-07

**Authors:** Mohammed S. Alharthi

**Affiliations:** Department of Clinical Pharmacy, College of Pharmacy, Taif University, Taif, Saudi Arabia

**Keywords:** challenges and enablers, electronic prescriptions, Wasfaty, Saudi Arabia, community pharmacists, theoretical domain framework (TDF)

## Abstract

**Background:**

Transition to electronic prescribing (e-prescribing) systems, such as Wasfaty, represents a significant advancement in healthcare. Introduced by the Saudi Arabian Ministry of Health in 2019, Wasfaty aims to enhance medication availability and streamline healthcare delivery. This study explores the challenges and enablers community pharmacists face when using the Wasfaty system in Saudi Arabia. This study uses the Theoretical Domains Framework (TDF) to analyse behavioural and contextual factors influencing pharmacists’ adoption of electronic prescriptions. TDF identifies key challenges and enablers across multiple behavioural domains, making it well-suited for understanding complex healthcare implementation processes.

**Methods:**

A qualitative study design was employed using the Theoretical Domains Framework (TDF) to understand factors affecting Wasfaty use. Participants were purposively sampled, focusing on community pharmacists experienced with Wasfaty prescriptions. Recruitment and interviews occurred from June to August 2024. Data saturation was achieved at 18 interviews. Transcripts were coded and mapped into TDF domains to identify barriers and enablers.

**Results:**

Five key TDF domains influenced Wasfaty use: environmental context and resources; social influences; beliefs about capabilities; social/professional role and identity; and knowledge. Challenges included high prescription volumes, medication shortages, technical difficulties, resistance from elderly patients, and inconsistent training. Some pharmacists reported inadequate training as a barrier, while others highlighted effective programs as enablers.

**Conclusion:**

Significant barriers, such as insufficient training and high prescription volumes, impede effective Wasfaty use. However, continuous training, prior experience with electronic systems, and organisational support were crucial enablers. Addressing these challenges through targeted interventions can enhance e-prescription efficiency, improving patient care and outcomes. Findings underscore the importance of ongoing professional development and supportive work environments in fostering pharmacists’ competence in electronic systems.

## Introduction

The transition to electronic prescribing systems, also known as e-prescriptions, represents a significant advancement in healthcare delivery, promising enhanced efficiency, accuracy, and safety in medication prescribing and dispensing (1, 2). Globally, several countries have successfully implemented electronic prescribing systems. For instance, the United Kingdom’s National Health Service (NHS) has developed a comprehensive e-prescribing system that facilitates the electronic transmission of prescriptions from primary care settings to community pharmacies (3). This system has been instrumental in reducing medication errors, improving prescription accuracy, and enhancing patient safety (4). Similarly, in the United States, the Electronic Prescribing for Controlled Substances (EPCS) system allows for the secure electronic transmission of controlled substance prescriptions, significantly reducing prescription fraud and abuse (5, 6). Countries such as Sweden and Denmark boast advanced e-prescribing systems, contributing to their efficient and high-quality healthcare services (7, 8).

In Saudi Arabia, the Ministry of Health introduced the Wasfaty system in 2019, an e-prescribing platform to modernise the nation’s healthcare infrastructure and improve patient outcomes. The Wasfaty electronic system was established to enhance the level of health services by ensuring the availability of medications. It links hospitals and primary healthcare centres with community pharmacies, making it easier for patients to receive their medications from the nearest community pharmacy (9). In Saudi Arabia, the use of the Wasfaty electronic prescribing system is mandatory for both doctors and pharmacies. Thousands of healthcare providers and pharmacies are currently using the system, ensuring widespread adoption across the country (10).

Electronic prescribing offers numerous advantages over traditional paper-based systems. It minimises medication errors, enhances the efficiency of the prescribing process, improves patient safety, and streamlines the workflow for healthcare providers (11). For patients, the Wasfaty system ensures that medications are more readily available and accessible. By linking healthcare providers directly with pharmacies, the system reduces patients’ time and effort obtaining prescriptions, thereby improving adherence to medication regimens. This accessibility is crucial for chronic patients requiring regular repeat medication (12, 13). Overall, e-prescribing systems such as Wasfaty can significantly improve quality of life by ensuring timely access to necessary medications, reducing the likelihood of treatment interruptions, and enhancing overall healthcare outcomes (14).

Research has identified common barriers to adopting e-prescribing systems across various contexts. These barriers include technical issues such as system downtimes and software incompatibilities, organisational challenges such as inadequate training and support, resistance to change, and workflow disruptions (15). Additionally, pharmacists may encounter individual-level barriers, including a lack of familiarity with the technology, concerns about increased workload, and apprehension regarding the reliability and security of electronic systems. Conversely, factors that can enable the successful adoption of e-prescribing systems include adequate training and support, positive perceptions of the system’s benefits, and the availability of resources to facilitate the transition (16). While prior research has highlighted the technical benefits and challenges of implementing e-prescribing systems globally, there is limited literature focusing on the behavioural and organisational challenges specific to Saudi Arabia’s context.

Implementing the Wasfaty system presents unique challenges and opportunities in Saudi Arabia. The country’s healthcare system is rapidly changing, with significant investments in digital health initiatives. However, adopting new technologies, particularly in community pharmacies, can be influenced by various cultural, organisational, and individual factors. For example, the level of digital literacy among pharmacists, the extent of support from healthcare authorities, and availability of the necessary infrastructure can all impact the uptake and use of e-prescribing systems.

The Wasfaty system aligns with Saudi Arabia’s Vision 2030, which aims to enhance the quality and efficiency of healthcare services by leveraging digital transformation to achieve world-class healthcare standards, thereby contributing to the overall improvement of public health (17, 18). By integrating advanced technologies such as the Wasfaty e-prescribing system, the Saudi government seeks to improve healthcare delivery, increase patient satisfaction, and ensure the sustainability of health services.

This study will contribute to the broader discourse on digital health transformation in Saudi Arabia. As the country strives to achieve its Vision 2030 goals, understanding the factors influencing the adoption of digital health technologies is essential. The findings from this study will inform strategies to support pharmacists in community pharmacies, ultimately contributing to the successful integration of e-prescribing systems and improving patient care.

This study aimed to explore the barriers and enablers that pharmacists face when dealing with electronic prescriptions in Saudi Arabian community pharmacies. By utilising the TDF, this research seeks to provide a nuanced and comprehensive understanding of the factors influencing pharmacists’ use of e-prescriptions. The findings will inform effective interventions and policies to support healthcare modernisation in Saudi Arabia, offering valuable insights into optimising the adoption and utilisation of e-prescribing systems, and fostering a more efficient and safe healthcare system.

## Methods

### Study design

This qualitative study used TDF to explore barriers and enablers facing community pharmacists dealing with electronic prescriptions (Wasfaty) conducted by the Ministry of Health. These domains were utilised to identify and understand the specific challenges and facilitators community pharmacists experienced in using electronic prescriptions.

### Recruitment

Pharmacists were recruited from community pharmacies involved with Wasfaty. Pharmacists in this study were recruited from multiple community pharmacy chains, ensuring a diversity of pharmacy sizes and operational practices were represented in the data. They were contacted via telephone or email and provided informed, written consent. Pharmacists were informed about the nature of the study. Inclusion criteria required pharmacists to have experience handling electronic prescriptions from hospitals and health centres in Makkah region of Saudi Arabia, and no minimum years of experience was required. Exclusion criteria included community pharmacies not dealing with electronic prescriptions, specifically Wasfaty, and pharmacists without experience in such prescriptions.

### Ethical approval

Ethical approval for the study was obtained from Taif University’s Ethics Committee application number 45-342, ensuring participant confidentiality and data protection throughout the research process.

### Sampling and sample size

Purposive sampling was employed to recruit 18 community pharmacists. Following the guidelines of Francis et al. (19), a minimum of 10 interviews were conducted initially, with further interviews carried out until data saturation was reached, resulting in 18 interviews.

### Interview procedure and data collection

Interview questions were developed based on the literature (20–22) and discussions with two experienced academics. The researcher, trained and experienced in qualitative interviews, conducted and audio-recorded the interviews, which were then transcribed verbatim. Participants provided demographic and professional information at the start of each interview, including gender, years of experience, position/role, educational background, and experience with e-prescribing systems. Interviews lasted between 30 min and 1 h, focusing on open-ended questions about the challenges and enablers faced by Wasfaty in daily practice. To minimize bias, a semi-structured interview guide was used to ensure consistency while allowing for in-depth exploration of participants’ experiences. Reflexivity was maintained by the interviewer to acknowledge potential biases during data collection.

### Data analysis

Interim analysis was conducted after the first 10 interviews, with coding continued until saturation at 18 interviews. Participant characteristics were summarised, and transcripts were manually coded into the 14 TDF domains. Each coded quote generated a belief statement grouped into domain. The researcher used memos and developed an audit trail to ensure trustworthiness, with verbatim quotes supporting the belief statements. The Theoretical Domains Framework (TDF) is a robust and integrative model synthesising multiple behaviour change theories. It is widely used in healthcare research to identify and understand factors influencing behaviour and inform intervention development. The TDF comprises 14 domains, including Knowledge, Skills, Social/professional role and identity, Beliefs about capabilities, Optimism, Beliefs about consequences, Reinforcement, Intentions, Goals, Memory, attention and decision processes, Environmental context and resources, Social influences, Emotion, Behavioural regulation. The TDF was selected over other theories as its domains are mapped to behaviour change techniques (BCTs), which are considered the active components of interventions, making it easier to choose effective elements for designing theory-based interventions aimed at changing practitioner behaviour (23). Furthermore, the TDF includes 128 theoretical constructs derived from 33 behaviour change theories, grouped into 14 domains (24–26). Applying the TDF allows this study to uncover the multifaceted barriers and enablers influencing pharmacists’ engagement with the Wasfaty system.

## Results

Table 1 shows the characteristics of 18 community pharmacists participating in the study. There were 11 males and seven females. The educational backgrounds varied, with 12 pharmacists holding a bachelor’s degree and six holding a PharmD. The years of experience as a pharmacist ranged from 1 to 10 years. Experience with electronic prescribing systems ranged from 0.6 to 4 years. Most pharmacists were involved in dispensing (15), with some also handling administrative duties (5).

**Table 1:** Characteristics of the community pharmacists who participated in the study.

Participant ID	Gender	Educational background	Years of experience as a pharmacist	Years of previous experience with E-prescribing systems	Position/role in the pharmacy
W1	Male	PharmD*	2.0	2.0	Dispensing, administrative
W2	Male	Bachelor	7.0	1.5	Dispensing
W3	Male	PharmD*	2.0	2.0	Dispensing
W4	Male	PharmD*	2.0	2.0	Dispensing
W5	Male	PharmD*	3.0	2.0	Dispensing
W6	Female	Bachelor	9.0	0.6	Dispensing
W7	Female	Bachelor	2.0	2.0	Dispensing
W8	Male	Bachelor	10.0	2.0	Dispensing
W9	Female	Bachelor	9.0	3.2	Dispensing, administrative
W10	Male	Bachelor	10.0	2.8	Dispensing, administrative
W11	Male	Bachelor	5.0	2.0	Dispensing
W12	Female	Bachelor	2.0	1.9	Dispensing
W13	Male	Bachelor	4.0	3.6	Dispensing
W14	Male	Bachelor	7.0	4.0	Dispensing
W15	Female	Bachelor	8.0	4.0	Dispensing
W16	Female	Bachelor	3.0	2.0	Dispensing, administrative
W17	Male	PharmD*	1.0	1.0	Dispensing
W18	Male	PharmD*	2.0	2.0	Dispensing, administrative

*PharmD, Pharmacy doctor.

### Community pharmacists’ challenges and enablers to electronic prescriptions (Wasfaty)

Based on the analysis of the interviews, the following TDF domains have been identified as key factors for using the Wasfaty system in community pharmacies in Saudi Arabia. The TDF domains identified in this study are:

1. Environmental context and resources.
2. Social influences.
3. Beliefs about capabilities.
4. Social/professional role and identity.
5. Skills.

### Environmental context and resources

One of the most significant issues reported by community pharmacists is the high volume of daily prescriptions. This high workload increases pressure on pharmacists, leading to potential errors and making it challenging to balance prescription handling with other pharmacy duties. This challenge is exacerbated by medication shortages due to supply chain issues or specific contracts with the Ministry of Health, which limit the options available for dispensing. Additionally, some pharmacists face difficulties navigating the Wasfaty system, mainly when prescription errors occur. The lack of a built-in electronic communication system necessitates direct contact with doctors, adding to the complexity and time required for managing prescriptions. Furthermore, community pharmacists reported that cancelling prescriptions and entering multiple prescription details was cumbersome and time-consuming, adding to the operational challenges pharmacists face.

"If there is an issue with a prescription, I have to contact the doctor directly because there's no built-in electronic communication system" W1.

"Managing a high volume of prescriptions daily, especially with the current shortages, becomes overwhelming, and the system doesn’t streamline the process efficiently." W3

On the other hand, supportive organisational policies and financial incentives have been highlighted as significant factors. These policies provide pharmacists with the tools and training to manage the Wasfaty system effectively. Additionally, pharmacies using the Wasfaty system generally experience better stock availability, which supports continuous drug supply and efficient pharmacy operations.

"We have financial incentives for handling a higher volume of electronic prescriptions" W7.

"Wasfaty system has contributed to increasing the availability of medications, especially chronic medications in large quantities" W1.

### Social influences

Social influences play a critical role in the adoption of the Wasfaty system. One major issue reported by community pharmacists is resistance from elderly customers, who often struggle with the electronic system and are resistant to switching medication brands when their preferred ones are out of stock. This issue is compounded when elderly customers send someone else to collect their prescriptions, making it challenging to resolve medication-related issues effectively. Additionally, communication with doctors presents a significant challenge, as pharmacists must clarify or correct prescriptions, which is time-consuming and adds to their workload. The timing of these communications can be problematic, as patients sometimes obtain the medication outside official working hours, resulting in delays in receiving responses from doctors.

"Sometimes the medication from certain companies runs out, and it becomes difficult to convince the customer to accept an alternative brand" W18.

"We also encounter issues with doctors when we need to clarify or correct doses. This is because the patients sometimes obtain the medication outside the official working hours for the doctors, and we may not get a response from them" W8.

Conversely, support from colleagues and supervisors significantly facilitates using the Wasfaty system. The presence of a supportive team helps pharmacists understand and troubleshoot the system more effectively. This supportive environment also helps overcome any challenges and improve overall workflow efficiency.

"Fortunately, my colleagues are very supportive and assist with understanding and troubleshooting the system" W3.

### Beliefs about capabilities

New pharmacists often lack confidence in managing the Wasfaty system, especially during the initial phase of their employment. This lack of confidence is primarily due to inexperience and unfamiliarity with the system. They may feel overwhelmed by the new technology and its demands, leading to hesitation and errors in prescription handling.

"Initially, there were some challenges with understanding the system and dealing with prescription cancellations" W17.

“I was unsure how to handle complex prescriptions at first, and I often doubted whether I was using the system correctly.” W12

However, this issue diminishes over time as pharmacists gain more experience and comprehensive training and develop confidence in managing electronic prescriptions effectively. Those with prior experience using electronic systems feel more capable and efficient in their roles. They become more adept at handling the system’s intricacies, which enhances their overall performance and reduces the likelihood of errors.

"I feel much more confident and efficient using the e-prescribing system now compared to when I first started" W2.

"With more practice and training, I’ve become comfortable navigating the system, and it no longer feels challenging." W9

### Social/professional role and identity

A clear understanding of roles within the pharmacy team and the responsibilities associated with using the Wasfaty system are significant factors. Pharmacists feel more confident and supported when their roles are well-defined and understood by their colleagues and supervisors. This clarity helps avoid role overlap and ensures that everyone in the team knows their specific duties related to electronic prescriptions. A well-defined professional identity enhances pharmacists’ job satisfaction and willingness to embrace new technologies.

"Well, it’s all bound to be a benefit. If everyone is communicating and everyone knows what each other is doing, it’s bound to be hugely beneficial for them" W16.

"When everyone in the team knows their responsibilities, it creates a smoother workflow, and we can manage prescriptions more efficiently." W5

### Skills

A significant challenge reported by community pharmacists is that initially, they lacked the skills required to use the Wasfaty system effectively. Pharmacists, especially those new to the system, often struggle with understanding its functions and navigating its features. This learning curve can hinder their ability to manage electronic prescriptions efficiently and may lead to frustration and decreased job performance. Adequate initial training is essential to bridge this skills gap and ensure pharmacists can use the system proficiently.

"When I first started using Wasfaty, I found it quite challenging due to the lack of proper training and guidance" W5.

"Some of the system features, like prescription adjustments and cancellations, were confusing at first because I didn’t have prior experience with such digital tools." W7

Conversely, some community pharmacists reported that they received training before working with Wasfaty. This training plays a crucial role in overcoming these challenges. Comprehensive training programmes and continuous learning opportunities help pharmacists acquire the necessary skills to use the system competently. The support from colleagues further enhances their understanding and capabilities. Ongoing education ensures pharmacists stay updated on system changes or new features, maintaining proficiency.

"The availability of foundational training courses provided by the organisation also contributes significantly to building my skills" W18.

"With ongoing practice and support, I gradually became more skilled and confident in using the system." W9

## Discussion

### Main finding

This study represents a pioneering effort to investigate the challenges and enablers of electronic prescriptions (Wasfaty) in Saudi Arabia using the TDF. The TDF identified five key domains influencing pharmacists’ engagement with the Wasfaty system: environmental context and resources, social influences, beliefs about capabilities, social/professional role and identity, and skills. These domains provided a comprehensive understanding of the challenges and enablers impacting the adoption and effective use of the Wasfaty system. The key findings underscore the significant hurdles pharmacists encounter initially, primarily stemming from a lack of comprehensive training on the Wasfaty system. This training gap leads to system comprehension and navigation difficulties, impeding efficiency and causing frustration. Many pharmacists feel overwhelmed by the system’s demands, adversely affecting their job performance.

Furthermore, the study underscores the positive influence of prior experience and ongoing training on pharmacists’ confidence and efficiency in using the Wasfaty system. Pharmacists with a history of working with electronic systems feel more competent and effective in their roles. Continuous training and support from colleagues are also pivotal in overcoming initial challenges, enabling pharmacists to master the system’s complexities, reduce errors, and enhance overall performance.

### Main discussion

A significant challenge to the efficient use of the Wasfaty system is the lack of skills among pharmacists. This is primarily due to insufficient formal training programmes provided by healthcare authorities or pharmacies. As a result, inexperienced users often rely on their own initiative or informal peer support to learn the system. Without structured training, pharmacists find it difficult to understand the system’s functions, navigate its features, and manage electronic prescriptions effectively. This lack of preparedness leads to feelings of being overwhelmed and lowers their confidence, especially among new pharmacists. Inadequate training also increases the likelihood of errors, which exacerbates frustration during the initial adoption phase. Similar findings have been reported in other studies where inadequate training was identified as a major barrier to adopting electronic health systems (27).

The study revealed variability in training provided to new pharmacists. While some received no training, others had only general pre-training, not specific to the Wasfaty system. This highlights the need for standardised, system-specific training to enhance the effective use of electronic prescribing in community pharmacies. On the other hand, pharmacists who received comprehensive training and had prior experience with electronic systems reported higher confidence and efficiency in their roles. This indicates that structured, continuous training is essential for equipping pharmacists with the skills needed to manage electronic prescriptions effectively.

Pharmacists who have a better understanding of the system’s functionalities and troubleshooting techniques are more likely to navigate the system seamlessly and reduce errors. This efficiency is enhanced by ongoing professional development and learning opportunities, which keep pharmacists informed about system changes and new features. A collaborative work environment where colleagues support one another further enhances the successful implementation of the system. These findings align with research by King et al. (28), which emphasised the importance of ongoing training and support in improving user confidence and proficiency.

The role of organisational support and infrastructure also emerged as critical in successfully implementing the Wasfaty system. Pharmacies that provide better organisational support, including policies, resources, and financial incentives, are more likely to encourage the adoption of new systems. This support helps in reducing resistance to change and improving overall efficiency. Institutional backing, such as financial incentives and supportive policies, creates an environment that promotes the successful adoption of new technologies. This finding is consistent with the work of Fenelly et al. (29), who highlighted the importance of organisational resources in integrating electronic prescribing systems.

Social influences also played a significant role in the adoption of the Wasfaty system. Resistance from older patients and communication challenges with doctors were notable obstacles. Older patients tend to be less familiar with technology and often resist change, preferring traditional methods. This resistance can hinder the seamless integration of the Wasfaty system. Furthermore, older patients usually trust their doctors more than pharmacists, which poses a challenge since the system often requires pharmacists to make adjustments to medications. Communication gaps between pharmacists and doctors can further delay and complicate the prescription process, highlighting the need for better integration within healthcare systems. Wilson et al. (30) discussed similar challenges faced by older adults in adopting electronic health records.

In contrast, support from colleagues and supervisors significantly facilitated the use of the system. Pharmacists who received encouragement and assistance from their peers felt more confident in navigating the system. A supportive team can provide valuable guidance and troubleshooting help, making the transition to electronic prescriptions smoother. This collaborative environment fosters continuous learning, which is essential for the successful implementation of new technologies. This finding is in line with previous research that emphasised the importance of a supportive work environment in adopting health information technologies (31).

Additionally, in comparison to the challenges faced in Saudi Arabia with the Wasfaty system, such as insufficient training and technical issues, countries like the UK (NHS) and the US (EPCS) have implemented electronic prescribing systems that have led to a reduction in prescription errors and improved patient safety (4, 6). While pharmacists in Saudi Arabia struggle with system downtimes and high prescription volumes, e-prescribing systems in Sweden and Denmark offer optimised, user-friendly interfaces that enhance workflow efficiency (28, 29). These international experiences emphasise the need for structured training and infrastructure improvements to ensure the effectiveness.

Globally, electronic prescribing systems have encountered similar challenges. For example, a study in Australia reported that insufficient communication between doctors and pharmacists led to delays in medication delivery and prescription errors (32). In response, many countries have adopted integrated systems that allow pharmacists and healthcare providers to communicate directly through the e-prescribing platform, reducing the need for external calls or follow-ups. Implementing similar measures in Saudi Arabia could help mitigate the communication gaps observed in the Wasfaty system.

When examining electronic prescribing systems across the Gulf region, significant variations emerge in implementation and integration. The Wasfaty system, introduced in Saudi Arabia, focuses on linking healthcare providers and community pharmacies to streamline prescription fulfilment and improve medication accessibility (10). In contrast, the United Arab Emirates’s system is integrated with a nationwide health information exchange, allowing for a more cohesive flow of patient data across healthcare facilities, thus reducing prescription errors and improving coordination (33). Similarly, Qatar’s e-prescribing system is embedded within its national healthcare platform, ensuring efficient and accurate medication dispensing (34).

### Strengths and limitations

One of the strengths of this study is the use of the TDF to explore the multifaceted barriers and enablers influencing pharmacists’ engagement with the Wasfaty system. The TDF is a robust and integrative model synthesising multiple behaviour change theories, making it particularly suitable for identifying and understanding factors influencing behaviour in healthcare settings (23). This approach provided a comprehensive understanding of the challenges and enablers, highlighting the importance of training, support, and organisational infrastructure. The sample size of 18 pharmacists is appropriate for a qualitative study of depth and detail, allowing for rich, detailed data collection.

However, the study has some limitations. While the sample size of 18 pharmacists is suitable for qualitative research, future studies should consider including larger and more diverse samples to increase the universality of the results. Another limitation of this study is that it was conducted solely in the Makkah region, which may limit the generalizability of the findings to other regions.

Additionally, the study relied on self-reported data from pharmacists, which may introduce bias. Therefore, incorporating the perspectives of other stakeholders, such as patients and doctors, would provide a more comprehensive understanding of the challenges and enablers associated with the Wasfaty system. This multifaceted approach could validate the findings and offer a more holistic view of the system’s impact on healthcare practice.

## Conclusion

The findings of this study have several important implications. First, there is a clear need for comprehensive and continuous training programmes to equip pharmacists with the skills required to manage electronic prescriptions. These programmes should include support from colleagues to further enhance understanding. Second, organisational support, including policies and infrastructure, plays a crucial role in the successful adoption of the Wasfaty system. Future research should explore the impact of these factors across different regions and settings to enhance the generalizability of the findings.

## Data Availability

The raw data supporting the conclusions of this article will be made available by the authors, without undue reservation.
